# An Online Prediction Method for Transient Frequency Response in New Energy Grids Based on Deep Integration of WAMS Data and Physical Model

**DOI:** 10.3390/e27111145

**Published:** 2025-11-10

**Authors:** Kailin Yan, Yi Hu, Han Xu, Tao Huang, Yang Long, Tao Wang

**Affiliations:** 1 School of Electrical Engineering and Electronic Information, Xihua University, Chengdu 610039, China; yankailin@stu.xhu.edu.cn (K.Y.); xuduidui@stu.xhu.edu.cn (H.X.); wangatao@163.com (T.W.); 2 Department of Energy, Politecnico di Torino, 10129 Turin, Italy; tao.huang@polito.it; 3 School of Electrical Engineering, Southwest Jiaotong University, Chengdu 611756, China; zhouchuanjiang@stu.xhu.edu.cn

**Keywords:** new energy grid, transient frequency prediction, WAMS data, information entropy, physics-guided learning, MLP-GRU-Attention model

## Abstract

The integration of a high proportion of renewable energy has significantly reduced the grid inertia level and markedly increased the risk of transient frequency instability in power systems. Meanwhile, the large-scale integration of diverse heterogeneous resources—such as wind power, photovoltaics, energy storage, and high voltage direct current (HVDC) transmission systems—has considerably enriched the portfolio of frequency regulation assets in modern power grids. However, the marked disparities in the dynamic response characteristics and actuation speeds among these resources introduce significant nonlinearity and high-dimensional complexity into the system’s transient frequency behavior. As a result, conventional methods face considerable challenges in achieving accurate and timely prediction of such responses. However, the substantial differences in the frequency regulation characteristics and response speeds of these resources have led to a highly nonlinear and high-dimensional complex transient frequency response process, which is difficult to accurately and rapidly predict using traditional methods. To address this challenge, this paper proposes an online prediction method for transient frequency response that deeply integrates physical principles with data-driven approaches. First, a frequency dynamic response analysis model incorporating the frequency regulation characteristics of multiple resource types is constructed based on the Single-Machine Equivalent (SME) method, which is used to extract key features of the post-fault transient frequency response. Subsequently, information entropy theory is introduced to quantify the informational contribution of each physical feature, enabling the adaptive weighted fusion of physical frequency response features and Wide-Area Measurement System (WAMS) data. Finally, a physics-guided machine learning framework is proposed, in which the weighted physical features and the complete frequency curve predicted by the physical model are jointly embedded into the prediction process. An MLP-GRU-Attention model is designed as the data-driven predictor for frequency response. A physical consistency constraint is incorporated into the loss function to ensure that predictions strictly adhere to physical laws, thereby enhancing the accuracy and reliability of the transient frequency prediction model. Case studies based on the modified IEEE 39-bus system demonstrate that the proposed method significantly outperforms traditional data-driven approaches in terms of prediction accuracy, generalization capability under small-sample conditions, and noise immunity. This provides a new avenue for online frequency security awareness in renewable-integrated power systems with multiple heterogeneous frequency regulation resources.

## 1. Introduction

In recent years, the penetration of renewable energy generation has continuously increased, while the proportion of conventional synchronous generators in the installed capacity continues to decline. Renewable energy generation units, such as wind and photovoltaic power systems, are typically integrated via power electronic interfaces. Their output power is decoupled from the system frequency, thus unable to provide inherent inertial support to the grid [1,2,3]. With the continuous increase in the penetration of renewable energy generation, the system’s equivalent inertia has significantly decreased, resulting in a higher Rate of Change of Frequency (RoCoF). During power disturbances, this leads to more severe transient frequency fluctuations, which increases the risk of triggering security control measures such as Under-Frequency Load Shedding (UFLS), thereby seriously jeopardizing the secure and stable operation of the power system [4,5,6].

Meanwhile, to mitigate the inherent variability and uncertainty of renewable energy and enhance renewable energy integration, new power electronic devices such as energy storage systems and HVDC transmission have been deployed on a large scale in power grids [7,8]. As a result, modern renewable-rich power systems incorporate various heterogeneous frequency regulation resources, including wind power, photovoltaics, energy storage, and HVDC technologies. While these resources enhance the system’s regulatory capacity, their frequency regulation characteristics, such as response speed, duration, and actuation mechanisms, differ significantly [9,10]. The coordination and cooperation among various resources are highly complex, resulting in a transient frequency response process characterized by strong nonlinearity, high-dimensional complexity, and significant uncertainty, thereby further increasing the difficulty of accurately predicting transient frequency.

Against this backdrop, achieving fast and accurate online prediction of transient frequency response has become a critical prerequisite for deploying emergency control strategies and ensuring the secure and stable operation of power grids [11]. Currently, methods for analyzing post-disturbance frequency responses in power systems can be broadly categorized into physics-based models and data-driven approaches [12]. Physics-based modeling methods, such as time-domain simulation [13] and equivalent model-based approaches [14], offer clear physical mechanisms, but these methods exhibit inherent limitations: time-domain simulation is computationally intensive and often unsuitable for online applications; although equivalent model-based approaches improve efficiency through simplification, they struggle to accurately capture the complex dynamic interactions among multiple heterogeneous resources, resulting in limited model accuracy.

Data-driven approaches offer novel solutions to the aforementioned challenges [15,16,17,18,19,20,21]. Reference [15] proposed a method for predicting power system frequency response (SFR) patterns after large disturbances based on a Deep Belief Network (DBN), enabling rapid frequency security assessment. Reference [16] developed an SFR model using deep transfer learning for wind-thermal hybrid power systems and obtained key parameters of the SFR model via an improved Recurrent Neural Network (RNN) to achieve frequency response prediction based on an equivalent model method. Reference [17] introduced a Frequency Security Predictor (FSPM) based on a one-dimensional Convolutional Neural Network (1D-CNN) and a temporal-feature attention module (TFAM), enabling simultaneous prediction of both Frequency Danger Level (FDL) and Time Security Margin (TSM). Reference [18] employed an attention-based Long Short-Term Memory (LSTM) neural network to predict grid frequency curves and used the Whale Optimization Algorithm (WOA) to optimize network parameters for improved prediction accuracy. Reference [19] applied a pruned one-dimensional time-aware CNN to analyze power system frequency stability and determine the optimal frequency regulation cost. Reference [20] implemented real-time grid frequency monitoring using a machine learning technique combining Nonlinear Auto Regressive (NAR) neural networks and LSTM networks based on wide-area measurement data. However, these methods heavily rely on large amounts of high-quality training data, exhibit “black-box” characteristics, suffer from limited generalizability and interpretability, may produce predictions that violate physical laws, and lack reliability in extreme disturbance scenarios with scarce data.

Given the respective limitations of physical models and data-driven methods, integrating the two approaches to leverage their respective strengths has emerged as a promising pathway to overcome current technical bottlenecks [22,23,24]. Based on real-time WAMS data, this paper proposes an online prediction method for transient frequency response that deeply integrates information entropy weighting and physics-guided learning. The main contributions of this paper are as follows:

(1) A physics-based frequency response analysis model incorporating the frequency regulation characteristics of multiple heterogeneous resources is developed to provide critical physical features for data-driven approaches.
(2) Information entropy theory is introduced to perform quantitative assessment and adaptive weighting of physical features, thereby enhancing the contribution of critical features in the model input.
(3) A physics-guided machine learning framework is proposed, which integrates the weighted physical features along with the complete frequency curve predicted by the physical model into the prediction process.
(4) An MLP-GRU-Attention hybrid model is designed as the data-driven engine, and a physical consistency constraint is incorporated into the loss function, thereby ensuring that the prediction results strictly adhere to physical laws.

The remainder of this paper is organized as follows: Section 2 establishes a physics-based frequency response model for renewable-integrated power systems and derives the calculation method for key features. Section 3 elaborates on the architecture of the prediction model, which integrates information entropy weighting and a physics-guided MLP-GRU-Attention framework. Section 4 introduces the offline training and online prediction workflow of the proposed method. Section 5 validates the superiority of the approach through case studies on a modified IEEE 39-bus system. Section 6 concludes the paper.

## 2. Physical Model Analysis of Dynamic Frequency Response in New Energy Grids

### 2.1. New Energy Grid Frequency Response Model

**Figure 1:**
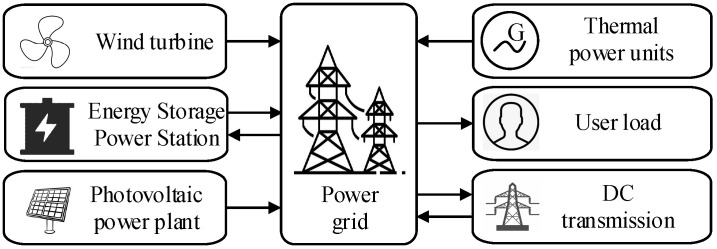
Schematic diagram of new energy grid.

Focusing on a typical renewable-energy integrated power system as illustrated in Figure 1, this paper takes into account the combined influence of conventional thermal units, renewable energy sources, energy storage systems, HVDC transmission, and load demand on frequency dynamics. An aggregate modeling approach is adopted to develop a physical frequency response model that incorporates multi-resource frequency regulation [14,25]. The dynamic responses of heterogeneous frequency regulation resources—including wind power, photovoltaic systems, energy storage, and HVDC transmission—are aggregated and represented by a low-order equivalent model. The frequency regulation control parameters of this equivalent model can be identified from actual measurement data.

(1) Frequency Response Model for Conventional Thermal Power Units

The frequency response characteristics of conventional thermal power units are primarily determined by the swing equation of synchronous generators and the dynamic characteristics of their governor systems [26]. Following a power disturbance after a system fault, the swing equation of the synchronous generator is described by

(1) $$2H\frac{d\Delta f}{dt}=\Delta P_{m}-\Delta P_{e}-D\Delta f$$

where H denotes the inertia time constant; $\Delta P_{m}$ and $\Delta P_{e}$ represent the variations in mechanical power and electromagnetic power, respectively; and D is the damping coefficient.

At the instant of disturbance, the change in electromagnetic power $\Delta P_{e}$ primarily originates from the external disturbance power $\Delta P_{d}$, which can be expressed as

(2) $$\Delta P_{e}=\Delta P_{d}$$

During the primary frequency regulation process, the governor adjusts the mechanical power through droop control:

(3) $$\Delta P_{m}=-K_{G}\Delta f$$

where $K_{G}$ is the inverse of the generator’s droop coefficient.

Substituting Equations (2) and (3) into Equation (1) yields the system frequency response equation:

(4) $$\frac{2H\Delta f}{dt}+(K_{G}+D)\Delta f=-\Delta P_{d}$$

Based on the equations presented above, the frequency response transfer function for the single-machine system can be derived as follows:

(5) $$\frac{\Delta f\left(s\right)}{\Delta P_{d}\left(s\right)}=-\frac{1}{2Hs+D+K_{G}}$$

**Figure 2:**
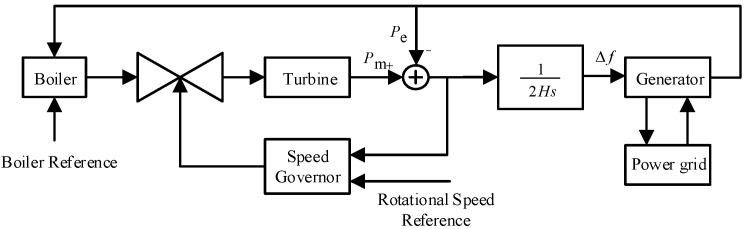
Frequency regulation model of conventional thermal power units.

The frequency regulation model of a conventional thermal power unit in the power system is illustrated in Figure 2. To improve computational efficiency and meet the real-time requirements for online frequency prediction, the original model, which includes components such as reheat turbines, has been appropriately simplified while preserving its essential dynamic characteristics. Certain nonlinearities and slow dynamic processes are neglected, with only the key primary frequency regulation and inertial response features retained.

**Figure 3:**
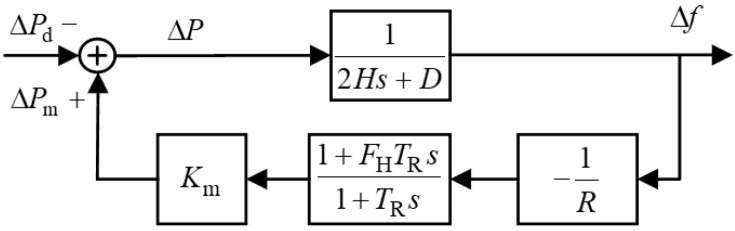
Single-machine equivalent frequency response model of the power system, where $\Delta P_{d}$ denotes the external disturbance power; $\Delta P_{m}$ represents the primary frequency regulation power of the synchronous generators; ΔP corresponds to the system accelerating power; $K_{m}$ is the gain coefficient; $F_{H}$ indicates the ratio of high-pressure turbine output power to the total mechanical power; and $T_{R}$ signifies the reheat time constant.

For practical multi-machine systems, the process of synchronous oscillation power exchange between units is neglected, and an equivalent aggregate unit is employed to represent the system’s average frequency response. By aggregating the swing equations and governor dynamics of all units into a single unified model, the overall representation focuses solely on power-frequency range responses, while neglecting slower dynamics such as boiler systems. This leads to the frequency response model of a multi-thermal-unit power grid, as illustrated in Figure 3.

(2) Frequency Response Characteristics of Wind Turbine Generators

Currently, grid-connected wind turbines widely employ grid-following converters, which inherently lack the ability to autonomously establish grid voltage and frequency and cannot provide natural inertial support like synchronous machines. To address this, frequency-regulating wind turbines typically adopt synthetic inertia control strategies that emulate the frequency regulation behavior of synchronous generators. This is achieved by incorporating virtual inertia control and primary frequency regulation into the wind turbine control system, including the release of rotational kinetic energy stored in the wind turbine rotor to provide inertial support, and the utilization of reserve capacity to deliver primary frequency regulation [27]. The virtual inertial response power of wind turbines can be described as follows:

(6) $$P_{\mathrm{wd}}=-k_{d}\frac{d\Delta f}{dt}$$

where $k_{d}$ is the virtual inertia time constant and Δf is the system frequency deviation.

The power increment of wind turbine units participating in primary frequency regulation can be represented as follows:

(7) $$P_{\mathrm{wp}}=-k_{p}\Delta f$$

where $k_{p}$ is the proportional parameter.

**Figure 4:**
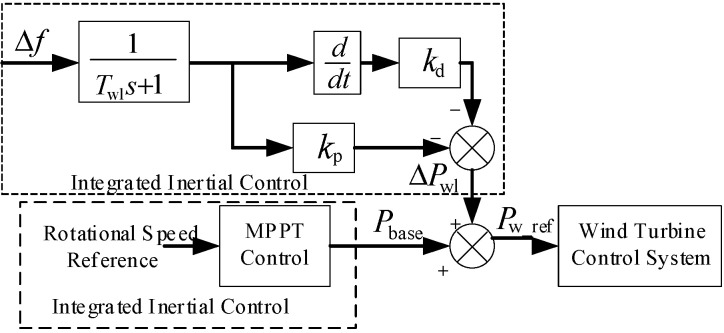
Frequency response model of wind turbine generator system.

Therefore, the synthetic inertia control strategy model for frequency-regulating wind turbines is developed, as illustrated in Figure 4.

In Figure 4, point $P_{\mathrm{base}}$ is set at the deloading operating point based on the system’s frequency regulation demand.

As can be seen from Figure 4, the output power $\Delta P_{\mathrm{wl}}$ of the synthetic inertia control strategy, accounting for the response time of wind turbine converters, can be expressed as follows:

(8) $$\Delta P_{\mathrm{wl}}=P_{\mathrm{wd}}+P_{\mathrm{wp}}=-\frac{k_{d}s+k_{p}}{T_{\mathrm{wl}}s+1}\Delta f$$

where $T_{\mathrm{wl}}$ denotes the frequency regulation time constant of the grid-following wind turbine, Δf represents the system frequency deviation, and s is the Laplace operator.

Therefore, the reference operating power $P_{w\_\mathrm{ref}}$ of a frequency-regulating wind turbine is obtained by combining the base operating power $P_{\mathrm{base}}$ during deloading operation with the additional frequency regulation power $\Delta P_{\mathrm{wl}}$ generated by virtual inertia and primary frequency regulation control, expressed as follows:

(9) $$P_{w\_\mathrm{ref}}=P_{\mathrm{base}}+\Delta P_{\mathrm{wl}}$$

In the equation, $P_{\mathrm{base}}$ represents the actual power setpoint of the wind turbine under deloaded operation, which is lower than the maximum capture power $P_{\mathrm{MPPT}}$ at the current wind speed. This ensures the wind turbine maintains upward reserve capacity. $\Delta P_{\mathrm{wl}}$ denotes the additional frequency regulation power, which includes both the reserve capacity activated by the wind turbine’s participation in primary frequency regulation control and the power sourced from the release of rotor kinetic energy through virtual inertial control, which refers to the conversion of kinetic energy into electrical energy by temporarily reducing rotor speed to provide rapid power support. To ensure that the wind turbine operates within its physical limits, the total output power $P_{\mathrm{base}}+\Delta P_{\mathrm{wl}}$ must always remain less than $P_{\mathrm{MPPT}}$.

(3) Frequency Response Characteristics of Photovoltaic (PV) and Energy Storage Systems

**Figure 5:**
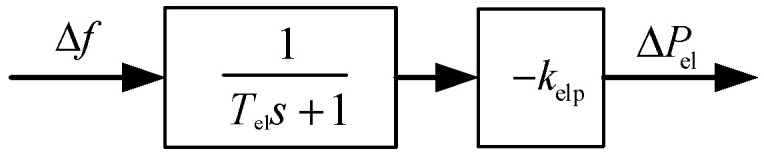
Frequency response model of PV and energy storage system.

PV power plants typically participate in grid frequency regulation through deloading operation or integrated energy storage systems. Given the inherent volatility and unpredictability of PV power generation, its rapid frequency response characteristics are comparable to those of energy storage systems. Therefore, this paper models the frequency regulation contributions of PV power plants and their associated energy storage systems in a unified manner [28,29]. Both PV plants and energy storage systems, which are typically grid-connected via power electronic converters, employ droop control strategies to provide primary frequency regulation. Their frequency response model is illustrated in Figure 5.

Therefore, the auxiliary frequency regulation output power $\Delta P_{\mathrm{el}}$ from the PV plant and the energy storage system can be expressed as follows:

(10) $$\Delta P_{\mathrm{el}}=-\frac{k_{\mathrm{elp}}}{T_{\mathrm{el}}s+1}\Delta f$$

where $k_{\mathrm{elp}}$ denotes the frequency droop control coefficient of the energy storage system, and $T_{\mathrm{el}}$ represents its control time constant.

(4) Frequency Response Characteristics of DC Transmission Systems

The HVDC transmission system, equipped with a Frequency Limiting Controller (FLC), can provide short-term transient frequency support for renewable-energy-integrated power grids, representing a form of transient ancillary service. The design objective of the FLC is to rapidly inject active power during the initial stage of a system disturbance when significant frequency deviation occurs, thereby restraining frequency changes. Its control strategy is centered on detecting when the system frequency deviation exceeds a preset dead band, upon which a proportional-integral (PI) controller is employed to generate an additional power command, dynamically adjusting the active power reference of the DC link [30].

**Figure 6:**
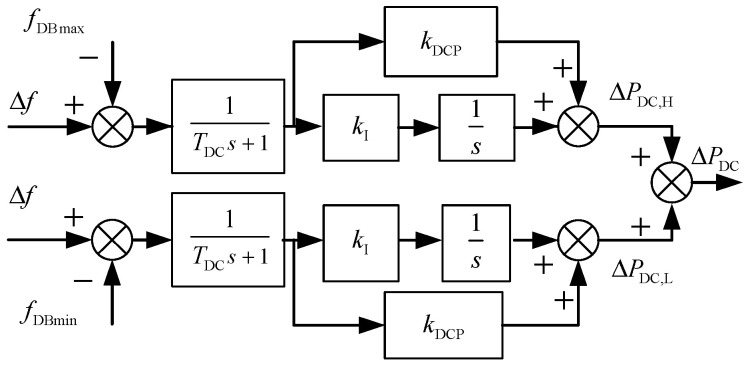
DC FLC frequency response model.

The FLC is not suitable for long-term operation under off-nominal DC voltage. To avoid conflicts with DC system protection settings and ensure long-term grid stability, the FLC typically adopts a reverse reset strategy. This strategy is activated once the frequency deviation returns within the dead band, gradually resetting the additional power reference to zero and restoring the DC system to its rated operating state. This mechanism effectively mitigates risks such as equipment overload, voltage limit violations, and maloperation of protection relays. The control structure is illustrated in Figure 6.

When the frequency deviation exceeds the control deadband, the positive and negative additional auxiliary power $\Delta P_{\mathrm{DC},H}$ and $\Delta P_{\mathrm{DC},L}$, as well as the total additional control regulatory signal $\Delta P_{\mathrm{DC}}$ of the HVDC transmission system, are given by the following:

(11) $$\left\{\begin{array}{l} \Delta P_{\mathrm{DC},H}=k_{\mathrm{DCP}}(\Delta f-f_{\mathrm{DBmax}})+\int k_{I}(\Delta f-f_{\mathrm{DBmax}})dt\\ \Delta P_{\mathrm{DC},L}=k_{\mathrm{DCP}}(\Delta f-f_{\mathrm{DBmin}})+\int k_{I}(\Delta f-f_{\mathrm{DBmin}})dt\\ \Delta P_{\mathrm{DC}}=\Delta P_{\mathrm{DC},H}+\Delta P_{\mathrm{DC},L} \end{array}\right.$$

where $f_{\mathrm{DB}\max}$ and $f_{\mathrm{DB}\min}$ represent the forward and reverse dead band values of the FLC control; $k_{\mathrm{DCP}}$ and $k_{I}$ denote the proportional and integral coefficients of the PI controller, respectively; and $T_{\mathrm{DC}}$ is the DC frequency regulation time constant. The proportional term $k_{\mathrm{DCP}}(\Delta f-\Delta f_{\mathrm{DB}\max})$ and the integral term $\int k_{i}(\Delta f-f_{\mathrm{DBmax}})dt$ in the equation correspond to the proportional control and integral control of the PI controller in Figure 6, respectively. As long as the frequency deviation is not completely eliminated, the integral term will continue to accumulate over time, thereby continuously adjusting the DC power reference value. This mechanism aims to ultimately eliminate steady-state frequency errors and ensure the accuracy of frequency recovery.

(5) Multi-Resource Frequency Response Model

Based on the frequency regulation models of the various resources described above, the corresponding transfer functions characterizing their frequency regulation properties are derived and summarized in Table 1.

**Figure 7:**
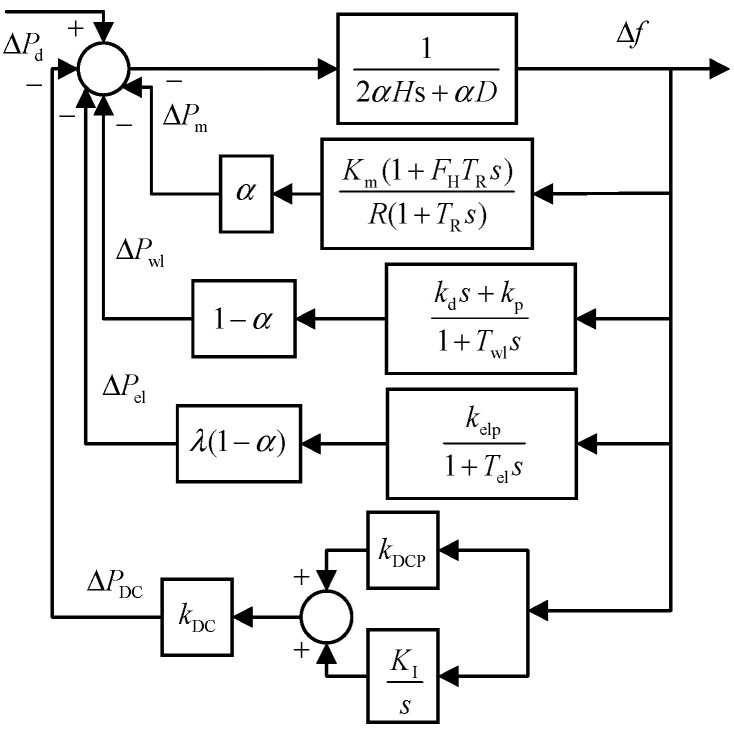
Frequency response model of new energy grid considering the participation of multiple frequency regulation resources.

Based on Table 1, the integrated frequency response model of the renewable-energy-integrated power system is constructed using the transfer function aggregation method, with its structure shown in Figure 7 [31]. The first branch represents the equivalent transfer function of the system rotor motion equation, characterizing the overall inertial response and damping characteristics of the system. The subsequent four branches correspond to the transfer function models of the synchronous generator governor, wind turbine virtual inertial control, photovoltaic and energy storage droop control, and HVDC FLC, respectively.

Based on this framework, the system swing equation can be written as follows:

(12) $$(2\alpha Hs+\alpha D)\Delta f=\Delta P_{d}-\Delta P_{m}-\Delta P_{\mathrm{wl}}-\Delta P_{\mathrm{el}}-\Delta P_{\mathrm{DC}}$$

Accordingly, the Laplace-domain expression of the frequency response in the renewable-integrated power system is derived based on Table 1 as follows:

(13) $$\Delta f=\frac{\Delta P_{d}}{2\alpha H+\alpha D+\frac{\alpha K_{m}(1+F_{H}T_{R}s)}{R(1+T_{R}s)}+(1-\alpha )\frac{k_{d}s+k_{p}}{1+T_{\mathrm{wl}}s}+\frac{(1-\alpha )\lambda k_{\mathrm{elp}}}{1+T_{\mathrm{el}}s}+k_{\mathrm{DC}}(k_{\mathrm{DCP}}+\frac{K_{I}}{s})}$$

where α denotes the proportion of synchronous generator capacity to the total frequency regulation resource capacity in the system, quantifying the contribution weight of synchronous units in the frequency response; λ represents the ratio of the capacity of the energy storage system coupled with wind turbines to the total wind power capacity, reflecting the enhancement degree of energy storage to the frequency regulation capability of wind turbines. Both are capacity proportion coefficients determined based on the actual configuration of the system’s frequency regulation resources, and their specific values can be obtained through statistical calculation of system parameters and resource capacity.

(14) $$\Delta f_{\mathrm{eq}}\approx \frac{R_{\mathrm{eq}}}{D_{\mathrm{eq}}R_{\mathrm{eq}}+K_{\mathrm{meq}}}\frac{(1+T_{\mathrm{Req}}s)\Delta P_{\mathrm{deq}}}{s(s^{2}+2\varsigma \omega_{n}+\omega_{n}^{2})}$$

(15) $$\omega_{n}^{2}=\frac{D_{\mathrm{eq}}R+K_{m}}{2H_{\mathrm{eq}}RT_{R}}$$

(16) $$\xi =\omega_{n}(\frac{2H_{\mathrm{eq}}R+D_{\mathrm{eq}}RT_{R}+K_{m}F_{H}T_{R}}{2(D_{\mathrm{eq}}R+K_{m})})$$

(17) $$H_{\mathrm{eq}}=2\alpha H+(1-\alpha )k_{d}$$

(18) $$D_{\mathrm{eq}}=\alpha D+(1-\alpha )k_{p}+(1-\alpha )\lambda k_{\mathrm{elp}}+k_{\mathrm{DC}}k_{\mathrm{DCP}}$$

Then, by applying the inverse Laplace transform, the time-domain analytical solution of the frequency response in the renewable-integrated power system incorporating multi-resource frequency regulation is obtained as follows:

(19) $$\begin{array}{ll} \Delta f_{\mathrm{eq}}(t) & =\frac{R\Delta P_{d}}{D_{\mathrm{eq}}R+K_{m}}[1-e^{-\xi \omega_{n}t}\cos(\sqrt{1-\xi^{2}}\omega_{n}t)\\ & -\frac{\xi -\omega_{n}T_{R}}{\sqrt{1-\xi^{2}}}e^{-\xi \omega_{n}t}\sin(\sqrt{1-\xi^{2}}\omega_{n}t)] \end{array}$$

### 2.2. Key Characteristics of Frequency Response in New Energy Grids

Based on the time-domain analytical solution of the SFR described in Equation (17), five key transient frequency response features with clear physical interpretations and computational tractability can be derived. These features concisely yet comprehensively characterize the frequency dynamics following a disturbance, and serve as essential inputs for the subsequent deep integration of WAMS data and physical knowledge. They include, but are not limited to, the following:

(1) Rate of Change of Frequency, RoCoF $\Delta f_{\mathrm{begin}}$

This characteristic reflects the initial rate of frequency decline following a disturbance and serves as a critical indicator for assessing the system inertia level. By taking the partial derivative of Equation (17) with respect to time, the RoCoF can be obtained as follows:

(20) $$\begin{array}{l} \Delta f_{b}=\frac{d\Delta f_{eq}(t)}{dt}=\\ \frac{R\Delta P_{d}}{D_{\mathrm{eq}}R+K_{m}}\omega_{n}e^{-\xi \omega_{n}t}[\frac{1-\xi \omega_{n}T_{R}}{\sqrt{1-\xi^{2}}}\sin(\sqrt{1-\xi^{2}}\omega_{n}t)+\\ \omega_{n}T_{R}\cos(\sqrt{1-\xi^{2}}\omega_{n}t)] \end{array}$$

Evaluating at time *t =* 0 yields the initial RoCoF, denoted as $\Delta f_{\mathrm{begin}}$:

(21) $$\begin{array}{l} \Delta f_{\mathrm{begin}}={\left.\frac{d\Delta f(t)}{dt_{con}}\right|}_{t=0}=\\ \frac{R\Delta P_{d}}{D_{\mathrm{eq}}R+K_{m}}\sin\varphi \omega_{n}T_{R} \end{array}$$

(2) Minimum Time to Arrival $t_{\mathrm{nadir}}$

This characteristic represents the time taken for the frequency to decline to its nadir, reflecting the response speed of the system’s frequency regulation resources. The maximum frequency deviation occurs at time $t_{\mathrm{nadir}}$. Given that the frequency nadir necessarily corresponds to an extremum point, where the partial derivative of Equation (17) with respect to time equals zero (i.e., the moment corresponding to $d\Delta f_{\mathrm{eq}}/dt=0$), the expression for $t_{\mathrm{nadir}}$ can be derived as follows:

(22) $$t_{\mathrm{nadir}}=\frac{1}{\sqrt{1-\xi^{2}}\omega_{n}}\arctan\left(\frac{\sqrt{1-\xi^{2}}\omega_{n}T_{R}}{\xi \omega_{n}T_{R}-1}\right)$$

(3) Lowest frequency $\Delta f_{\min}$

This characteristic corresponds to the lowest point (nadir) reached during the frequency response process, which directly determines whether safety control measures such as UFLS will be triggered. Based on the provided analytical expression of the frequency response and the expression for the time of maximum frequency deviation $t_{\mathrm{nadir}}$, substituting $t_{\mathrm{nadir}}$ into the frequency response equation yields the maximum frequency deviation value:

(23) $$\begin{array}{ll} \Delta f_{\min} & =\frac{R\Delta P_{d}}{D_{\mathrm{eq}}R+K_{m}}[1-e^{-\xi \omega_{n}t_{\mathrm{nadir}}}\cos(\sqrt{1-\xi^{2}}\omega_{n}t_{\mathrm{nadir}})\\ & -\frac{\xi -\omega_{n}T_{R}}{\sqrt{1-\xi^{2}}}e^{-\xi \omega_{n}t_{\mathrm{nadir}}}\sin(\sqrt{1-\xi^{2}}\omega_{n}t_{\mathrm{nadir}})] \end{array}$$

(4) Steady-state frequency $f_{\mathrm{ss}}$

This characteristic signifies the frequency value at which the system settles into a new stable operating state following a disturbance, reflecting the capacity and droop characteristics of the frequency regulation resources. By taking the limit as $t_{con}$ approaches infinity, the steady-state frequency $f_{sta}$ can be calculated as follows:

(24) $$f_{\mathrm{ss}}=\underset{t\to \infty }{\lim}\Delta f_{eq}(t)=\frac{R\Delta P_{d}}{D_{\mathrm{eq}}R+K_{m}}$$

(5) Steady-State Frequency Settling Time $t_{\mathrm{ss}}$

This characteristic is defined as the time required for the SFR to enter and remain within a ±2% error band of the steady-state value. It reflects the speed of frequency recovery and can be expressed as follows:

(25) $$t_{\mathrm{ss}}=-\frac{1}{\xi \omega_{n}}\ln(\frac{0.02\sqrt{1-\xi^{2}}}{\sqrt{1-2\xi \omega_{n}T_{R}+{\left(\omega_{n}T_{R}\right)}^{2}}})$$

The key characteristic quantities derived from the analytical solution of the equivalent model form the physical information foundation for the subsequent physics–data hybrid prediction model. The values of these characteristic quantities are uniquely determined by the system’s equivalent parameter set, which essentially represents the comprehensive manifestation of the coordinated effects of operational conditions and all frequency regulation resource control systems. Therefore, the equivalent model developed in this study describes, through its identifiable equivalent parameters, the intrinsic properties of the system’s overall frequency response under given system structures and control strategies.

## 3. A Frequency Prediction Architecture Integrating WAMS Information with Physical Model

### 3.1. Principles of the MLP-GRU-Attention Model

**Figure 8:**
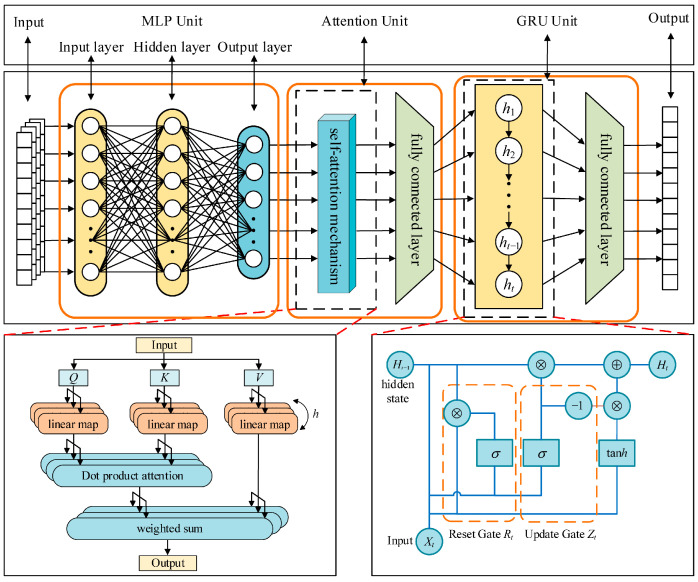
Schematic diagram of the MLP-GRU-Attention model structure.

To achieve accurate sequence prediction of power system transient frequency, a model capable of simultaneously processing high-dimensional spatial features, complex temporal dynamics, and maintaining physical interpretability must be constructed. For this purpose, this paper designs a hybrid MLP-GRU-Attention architecture, whose overall structure is illustrated in Figure 8. In this architecture, the MLP encoder is responsible for fusing and encoding the WAMS multi-bus measurement data and the key features from the physical model into a global feature vector. The GRU decoder is specifically designed to learn the complex temporal patterns arising from the dynamic responses of heterogeneous frequency regulation resources [32,33,34]. The attention mechanism dynamically focuses on the key features at different prediction stages during the decoding process. These three components work synergistically, forming a complete technical framework that progresses from “feature fusion” to “temporal learning” and finally to “dynamic focusing”, collectively enhancing the prediction accuracy and reliability for the entire transient frequency response process.

(1) Encoder Design

The encoder employs a Multilayer Perceptron (MLP) as its core structure. Its input is the preprocessed and feature-weighted fused vector $X=\left[X_{\mathrm{WAMS}},X_{\mathrm{phy}}\right]$, which consists of real-time grid snapshot data collected by WAMS—including node voltages (*V*), phase angles (*θ*), active power (*P*), and reactive power (*Q*)—and vector $X_{\mathrm{phy}}$, which represents the physically critical features weighted by information entropy, such as the initial RoCoF $\Delta f_{\mathrm{begin}}$, frequency nadir $\Delta f_{\min}$, and steady-state frequency $f_{\mathrm{ss}}$.

The MLP compresses and aggregates high-dimensional features through a three-layer nonlinear transformation, with the specific process described as follows:

(1) Input layer

The fused feature vector *X* is mapped into the hidden layer space, where its dimensionality is reduced from $d_{\mathrm{in}}$ to $d_{\mathrm{in}}$ using the ReLU activation function, as follows:

(26) $$H_{1}=\mathrm{ReLU}\left(W_{1}X+b_{1}\right)$$

where $W_{1}\in {\mathbb{R}}^{d_{h1}\times d_{\mathrm{in}}}$ and $b_{1}\in {\mathbb{R}}^{d_{h1}}$ denote learnable parameters.

(2) Hidden layer

The transformed features $H_{1}$ undergo further nonlinear transformation while maintaining the dimensionality $d_{h1}$, thereby enhancing the model’s capacity to capture complex nonlinear relationships. This process is expressed as follows:

(27) $$H_{2}=\mathrm{ReLU}\left(W_{2}H_{1}+b_{2}\right)$$

where $W_{2}\in {\mathbb{R}}^{d_{h1}\times d_{\mathrm{in}}}$ and $b_{2}\in {\mathbb{R}}^{d_{h1}}$ denote learnable parameters.

(3) Output layer

The input $H_{2}$ is encoded into a global feature vector $X_{\mathrm{ctx}}$, with its dimensionality compressed to $d_{\mathrm{ctx}}$. This vector comprehensively represents the initial state and correlations among key features following a grid disturbance, as expressed by the formula:

(28) $$X_{\mathrm{ctx}}=W_{3}H_{2}+b_{3}$$

where $W_{3}\in {\mathbb{R}}^{d_{h1}\times d_{\mathrm{in}}}$ and $b_{3}\in {\mathbb{R}}^{d_{h1}}$ denote learnable parameters.

(2) Decoder Design

The decoder employs a Gated Recurrent Unit (GRU) network to generate the future time-step frequency prediction sequence ${\hat{y}}_{1},{\hat{y}}_{2},\ldots ,{\hat{y}}_{T}$ in an autoregressive manner. By dynamically regulating the retention and forgetting of historical information through its update and reset gates, the GRU effectively mitigates the vanishing gradient problem during long-sequence training. The core computational procedure is as follows:

(1) Reset Gate $r_{t}$

It regulates the influence of the previous hidden state $h_{t-1}$ on the current candidate hidden state $h_{t}$, as expressed by the formula:

(29) $$r_{t}=\sigma \left(W_{r}\left[h_{t-1},{\hat{y}}_{t-1},X_{\mathrm{ctx}}\right]+b_{r}\right)$$

where σ denotes the Sigmoid activation function, $h_{t-1}\in {\mathbb{R}}^{d_{h2}}$ represents the hidden state from the previous time step, ${\hat{y}}_{t-1}$ corresponds to the predicted frequency value at the previous time step, and $W_{r}\in {\mathbb{R}}^{d_{h2}\times \left(d_{h2}+1+d_{\mathrm{ctx}}\right)}$ and $b_{r}\in {\mathbb{R}}^{d_{h2}}$ are learnable parameters.

(2) Update Door $z_{t}$

It controls the integration ratio between the historical hidden state $h_{t-1}$ and the current candidate hidden state $h_{t}$, as expressed by the formula:

(30) $$z_{t}=\sigma \left(W_{z}\left[h_{t-1},{\hat{y}}_{t-1},X_{\mathrm{ctx}}\right]+b_{z}\right)$$

where $W_{z}\in {\mathbb{R}}^{d_{h2}\times \left(d_{h2}+1+d_{\mathrm{ctx}}\right)}$ and $b_{z}\in {\mathbb{R}}^{d_{h2}}$ denote learnable parameters.

(3) Candidate Hidden Status ${\tilde{h}}_{t}$

The hidden state is updated based on the historical information filtered by the reset gate and the current input, as follows:

(31) $$\left\{\begin{array}{l} h_{t}=\left(1-z_{t}\right)⊙h_{t-1}+z_{t}⊙{\tilde{h}}_{t}\\ {\hat{y}}_{t}=W_{y}h_{t}+b_{y} \end{array}\right.$$

where ⊙ denotes the element-wise multiplication operation, $W_{\tilde{h}}\in {\mathbb{R}}^{d_{h2}\times \left(d_{h2}+1+d_{\mathrm{ctx}}\right)}$ and $b_{\tilde{h}}\in {\mathbb{R}}^{d_{h2}}$ represent learnable parameters.

(4) Current hidden state $h_{t}$ and predicted output ${\hat{y}}_{t}$

By combining the update gate weights, the current hidden state is generated and subsequently mapped to the frequency prediction value through a linear layer, as expressed by the formula:

(32) $$\left\{\begin{array}{l} h_{t}=\left(1-z_{t}\right)⊙h_{t-1}+z_{t}⊙{\tilde{h}}_{t}\\ {\hat{y}}_{t}=W_{y}h_{t}+b_{y} \end{array}\right.$$

where $W_{y}\in {\mathbb{R}}^{d_{h2}\times 1}$ and $b_{y}\in \mathbb{R}$ denote learnable parameters.

(3) Design of the Attention Mechanism

The attention mechanism serves as a critical component bridging the encoder and the decoder [35,36]. Its core function is to enable the decoder to dynamically focus on the features most relevant to the current prediction task within the global feature vector produced by the encoder when generating frequency predictions at each time step, thereby enhancing the model’s sensitivity to key features and improving predictive interpretability. The specific computational steps are as follows:

(1) Computation of Attention Scores

The attention score $e_{t}$ is computed based on the similarity between the hidden state $h_{t-1}$ from the previous decoder time step and the global feature vector $X_{\mathrm{ctx}}$ from the encoder, using the scaled dot-product method. The formula is given as follows:

(33) $$e_{t}=\frac{h_{t-1}^{T}X_{\mathrm{ctx}}}{\sqrt{d_{\mathrm{ctx}}}}$$

where $\sqrt{d_{\mathrm{ctx}}}$ is a scaling factor introduced to mitigate the gradient saturation issue in the Softmax function caused by excessively large values after the dot product of high-dimensional vectors.

(2) Normalization of Attention Weights

The attention scores are converted into normalized weights $\alpha_{t}$ through the Softmax function, ensuring the sum of the weights equals 1, as follows:

(34) $$\alpha_{t}=\frac{\exp\left(e_{t}\right)}{\sum_{k=1}^{K}\exp\left(e_{k}\right)}$$

where *K* denotes the number of segments of the global feature vector $X_{\mathrm{ctx}}$, which are partitioned according to physical feature categories such as $\Delta f_{\mathrm{begin}}$, $\Delta f_{\min}$, and $f_{\mathrm{ss}}$.

(3) Dynamic Global Feature Vector Generation

Based on the attention weights $\alpha_{t}$, a weighted sum of the global feature vector $X_{\mathrm{ctx}}$ is computed, yielding the dynamic global feature vector $x_{t}$ for the current time step, as follows:

(35) $$x_{t}=\sum_{k=1}^{K}\alpha_{t}^{k}X_{\mathrm{ctx}}^{k}$$

where $\alpha_{t}$ denotes the *k*-th segment of the global feature vector $X_{\mathrm{ctx}}$, and $x_{t}$ represents the corresponding attention weight for that segment.

### 3.2. Information Entropy-Based Weighting Method for Physical Features

The key features output by physical models exhibit varying information values across different grid disturbance scenarios. For instance, feature $\Delta f_{\min}$ demonstrates significant fluctuations during fault scenarios, containing rich transient stability information, whereas feature $f_{\mathrm{ss}}$ changes gradually in steady-state conditions, contributing less informational value. To quantify the information value of each physical feature and achieve adaptive weighting, this paper introduces information entropy theory, which characterizes the information content of a feature by its degree of uncertainty.

(1) Physical Feature Extraction

Based on the physics-based frequency response analysis model incorporating multi-resource frequency regulation developed in Section 1, the section data collected by WAMS is processed to compute and output *N* physical key features, forming the feature set $K_{N}=\left\{K_{1},K_{2},\ldots ,K_{n}\right\}$. To calculate the information entropy, this paper adopts the equal-frequency discretization method, dividing the value range of each feature $K_{i}$ into *m* intervals such that each interval contains approximately the same number of historical samples, thereby reducing discretization error. Let the discrete intervals of feature $K_{i}$ be denoted as $\left[x_{i1},x_{i2}\right)$, $\left[x_{i3},x_{i4}\right)$, …, $\left[x_{im},x_{i\left(m+1\right)}\right]$, where $x_{i1}$ and $x_{i\left(m+1\right)}$ represent the minimum and maximum values of the feature, respectively.

(2) Computation of Information Entropy and Weight Normalization

Information Entropy $H\left(K_{i}\right)$ is used to quantify the uncertainty of feature $K_{i}$ across different scenarios. A higher entropy value indicates a more dispersed distribution of the feature’s values, which corresponds to richer information content and a greater contribution to frequency prediction. Based on historical datasets, the probability $p_{ij}$ of feature $K_{i}$ falling into the *j*-th discrete interval (where *j* = 1, 2, …, *m*) is statistically derived. The information entropy $H\left(K_{i}\right)$ is calculated as follows:

(36) $$H\left(K_{i}\right)=-\sum_{j=1}^{m}p_{ij}\log_{2}p_{ij}$$

To address the issue of magnitude disparities in raw entropy values, a normalization procedure is applied to the information entropy of each feature, resulting in the feature weight $\omega_{i}$ and ensuring that the sum of all weights equals 1. The formula is defined as follows:

(37) $$\omega_{i}=\frac{H\left(K_{i}\right)}{\sum_{i=1}^{N}H\left(K_{i}\right)}$$

(3) Weighted Physical Feature Vector Formulation

Each physical feature $K_{i}$ is multiplied by its corresponding weight $\omega_{i}$ to obtain the weighted physical feature $K_{\mathrm{phy}}^{i}=K_{i}\cdot \omega_{i}$. These weighted features are then integrated to form the weighted physical feature vector $X_{\mathrm{phy}}=\left[K_{\mathrm{phy}}^{1},K_{\mathrm{phy}}^{2},\ldots ,K_{\mathrm{phy}}^{N}\right]$. This vector is concatenated with the raw WAMS snapshot data vector $X_{\mathrm{WAMS}}$, forming the input vector $X=\left[X_{\mathrm{WAMS}},X_{\mathrm{phy}}\right]$ for the MLP encoder. This process achieves a deep fusion of system-level physical priors with spatiotemporal multi-bus measurement data, establishing an informational foundation for subsequent data-driven models that incorporates both physical consistency and spatial detail.

### 3.3. Construction of the Weighted Physical Feature Vector

To further strengthen the integration of physical knowledge with data-driven models and prevent predictions that violate grid physical laws, including sudden frequency jumps or steady-state frequency deviations beyond acceptable ranges, this paper proposes a physics-guided fusion architecture. This framework incorporates a physics-informed hybrid loss function that embeds power system operational constraints into the training process, ensuring both high accuracy in fitting historical data and strict adherence to physical requirements.

Conventional data-driven models solely employ the Mean Squared Error (MSE) to quantify the deviation between predicted and true values, which can lead to predictions that violate physical laws. To address this issue, this paper designs a hybrid loss function *L*, comprising a data fitting loss term $L_{\mathrm{MSE}}$ and a physical consistency loss term $L_{\mathrm{phy}}$, formulated as follows:

(38) $$L=\alpha \cdot L_{\mathrm{MSE}}+\beta \cdot L_{\mathrm{phy}}$$

where the hyperparameters *α* and *β* are adjustable.

(1) Data Fitting Loss $L_{\mathrm{MSE}}$

This component measures the overall deviation between the model-predicted frequency sequence $\hat{Y}=\left[{\hat{y}}_{1},{\hat{y}}_{2},\ldots ,{\hat{y}}_{T}\right]$ and the true frequency sequence $Y=\left[y_{1},y_{2},\ldots ,y_{T}\right]$, ensuring the model’s baseline prediction accuracy. It is defined as follows:

(39) $$L_{\mathrm{MSE}}=\frac{1}{T}\sum_{t=1}^{T}{\left({\hat{y}}_{t}-y_{t}\right)}^{2}$$

(2) Physics Consistency Loss

The design of $L_{\mathrm{phy}}$ is grounded in the core physical principles governing power system transient frequency response, targeting the following three key metrics to formulate constraint terms:

(1) Initial RoCoF Consistency Constraint

This constraint ensures that the deviation between the predicted initial RoCoF $\Delta {\hat{f}}_{\mathrm{begin}}=\left({\hat{y}}_{2}-{\hat{y}}_{1}\right)/\Delta t$ and the value $\Delta f_{\mathrm{begin}}^{\mathrm{phy}}$ calculated by the physical model remains below a threshold $\epsilon_{1}=0.1$ Hz/s. It is formulated as follows:

(40) $$L_{\Delta f_{\mathrm{begin}}}=\max\left(0,\left|\Delta {\hat{f}}_{\mathrm{begin}}-\Delta f_{\mathrm{begin}}^{\mathrm{phy}}\right|-\epsilon_{1}\right)$$

(2) Frequency Nadir Consistency Constraint

This constraint ensures that the deviation between the predicted value $\Delta {\hat{f}}_{\min}=\min\left({\hat{y}}_{1},{\hat{y}}_{2},\ldots ,{\hat{y}}_{T}\right)$ and the value $\Delta f_{\min}^{\mathrm{phy}}$ calculated by the physical model remains below a threshold $\epsilon_{2}=0.05$ Hz, formulated as follows:

(41) $$L_{\Delta f_{\min}}=\max\left(0,\left|{\hat{f}}_{\Delta \min}-f_{\Delta \min}^{\mathrm{phy}}\right|-\epsilon_{2}\right)$$

(3) Steady-State Trend Consistency Constraint

This constraint ensures that the deviation between the predicted steady-state frequency ${\hat{f}}_{\mathrm{ss}}={\hat{y}}_{T}$ (assuming steady state is reached at time *T*) and the value $f_{\mathrm{ss}}^{\mathrm{phy}}$ calculated by the physical model remains below the threshold $\epsilon_{3}=0.02$ Hz, formulated as follows:

(42) $$L_{f_{\mathrm{ss}}}=\max\left(0,\left|{\hat{f}}_{\mathrm{ss}}-f_{\mathrm{ss}}^{\mathrm{phy}}\right|-\epsilon_{3}\right)$$

Thus, the comprehensive formula for the physics consistency loss $L_{\mathrm{phy}}$ is given by

(43) $$L_{\mathrm{phy}}=L_{\Delta {\hat{f}}_{\mathrm{begin}}}+L_{\Delta f_{\min}}+L_{f_{\mathrm{ss}}}$$

Through the aforementioned hybrid loss function, the model is required not only to fit historical data during training but also to satisfy physical constraints, thereby significantly enhancing the reliability and generalization capability of predictions. This approach is particularly suitable for scenarios where grid inertia fluctuates substantially due to high penetration of renewable energy integration.

## 4. Online Prediction Method for Transient Frequency Response Based on the Fusion of WAMS Data and Physical Model

**Figure 9:**
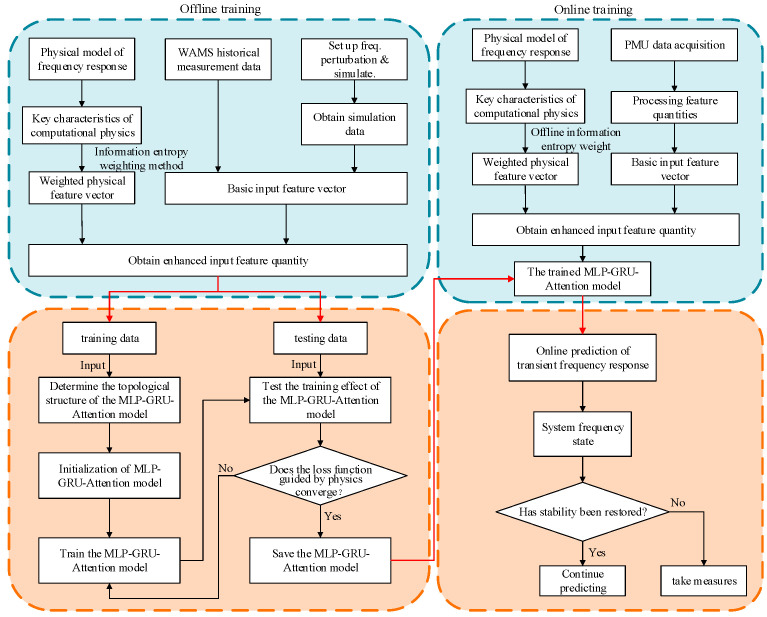
Flowchart of the online prediction method for transient frequency response based on WAMS data and physical model fusion.

Based on the frequency prediction architecture integrating WAMS information and physical model introduced in the previous section, this paper proposes an online prediction method for transient frequency response that fuses WAMS data with the physical model. The implementation process primarily consists of two key stages: offline training and online prediction. The specific workflow is illustrated in Figure 9.

(1) Offline Training

(1) Use historical measurement data from the renewable energy grid WAMS and time-domain simulation data from the frequency response physical model, incorporating multiple heterogeneous frequency regulation resources as raw sample data. Perform data cleaning, missing data repair, time alignment, and resampling on the raw samples to ensure temporal consistency and data integrity.

(2) Extract pre-disturbance steady-state grid cross-section data, instantaneous disturbance data, and post-disturbance initial-time data from the preprocessed WAMS historical measurement data and time-domain simulation data to form the basic input feature set.

(3) Based on the time-domain analytical solution for renewable grid frequency response and related formulas derived in Section 2.2, compute the physical key features for each sample. Combine these with the information entropy weighting method from Section 3.2 to obtain weighted physical feature vectors. Concatenate the basic input feature set with the weighted physical feature vectors to form the complete input set.

(4) Map the values of the complete input set to the interval [0, 1] for normalization. This eliminates interference from dimensional differences among features during model training, ensuring convergence efficiency.

(5) Randomly partition the normalized input set into training and testing sets at a 2:1 ratio. Configure the parameters of the MLP-GRU-Attention model.

(6) After initializing the MLP-GRU-Attention model parameters, feed the training set into the model and train using the hybrid loss function designed in Section 3.3. Continue training until the model’s loss value converges on the training set. If the prediction error on the test set does not meet the preset requirements, adjust the model hyperparameters. Iterate through the parameter tuning and model training process until the prediction error on the test set stabilizes within an acceptable range. This ultimately yields the fully trained physics-guided MLP-GRU-Attention frequency prediction model.

(2) Online Prediction

(1) WAMS data is collected in real time via phasor measurement units (PMU) extensively deployed across the grid and uploaded to the master station for rapid preprocessing.

(2) Based on real-time grid section characteristics, invoke the physical model equations from the offline training phase to rapidly compute real-time physical key features. Apply the offline-trained information entropy weights to these features, yielding a real-time weighted physical feature vector. Concatenate the real-time grid section characteristics with the weighted physical feature vector, normalize the combined input, and feed it into the offline-trained MLP-GRU-Attention frequency prediction model.

(3) The model outputs real-time transient frequency response prediction curves and key indicators. Based on the prediction results, it determines whether the system frequency is stable. If the frequency prediction indicates that the system can recover to stability, it continues collecting real-time data for subsequent frequency predictions. Otherwise, it immediately triggers emergency grid control measures to prevent system frequency instability.

## 5. Analysis of the Algorithm

### 5.1. Example System

**Figure 10:**
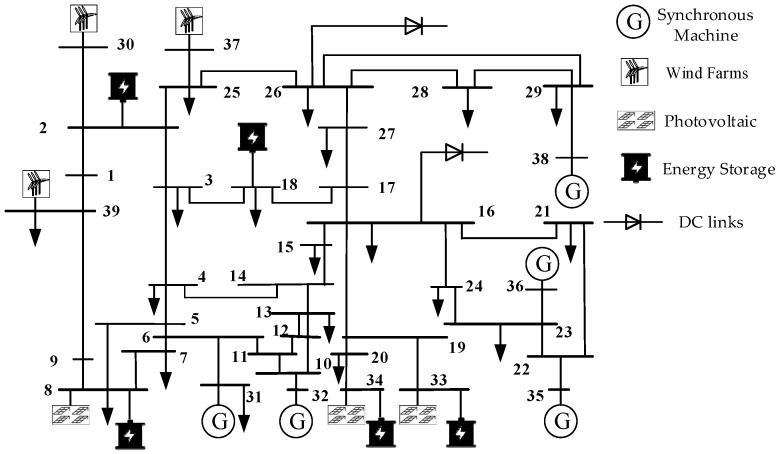
Improved IEEE39 node grid structure diagram.

To validate the effectiveness of the proposed method, this paper establishes a detailed time-domain simulation model in PSS/E that emulates a realistic renewable energy-integrated power grid. The pre- and post-disturbance data obtained from the time-domain simulations are utilized as the WAMS data required by the proposed approach. For this purpose, the conventional IEEE 39-bus system is modified by incorporating equivalent wind farms at buses 30, 37, and 39, equivalent photovoltaic power stations at buses 8, 33, and 34, and DC links at buses 16 and 26. This transforms the grid into a typical power system with high penetration of renewable energy, whose structure is illustrated in Figure 10.

The MLP-GRU-Attention model hyperparameters are set as follows: 2-layer MLP encoder and 1-layer GRU decoder structure, 4-head attention mechanism, hidden layer dimension 64, feed-forward network dimension 256 (ReLU activation function is used), batch size 32, initial learning rate 1 × 10^−4^ (Adam’s optimizer), Dropout rate 0.2, and number of training rounds, i.e., 150 rounds (with early stopping method). The input features are Min–Max normalized to the interval [0, 1].

In this paper, Python 3.9 programming is used to invoke the PSS/E simulation platform to carry out cyclic simulation analysis of the aforementioned new energy grid, where load increase perturbation is carried out for a single load node or multiple load nodes. The size of the load perturbation is randomly set in the range of 0%~50%, and the operation data of each node before and immediately after load perturbation is proposed to be the basic data of the WAMS, so as to obtain 9000 groups of samples, including 6000 groups of training samples and 3000 groups of testing samples.

### 5.2. Performance Evaluation Index

To comprehensively evaluate the performance of the proposed prediction model from multiple dimensions, this paper selects four evaluation metrics with complementary physical significance: Mean Absolute Error (MAE), Root Mean Square Error (RMSE), Coefficient of Determination (R^2^), and Dynamic Time Warping (DTW). These metrics provide quantitative assessments from the perspectives of absolute error level, sensitivity to large errors, goodness of trend fit, and transient process shape similarity, respectively. Their definitions are as follows:

(1) Mean Absolute Error (MAE)

MAE calculates the average of absolute differences between predicted values and true values, which can intuitively reflect the overall level of prediction errors.

(44) $$V_{\mathrm{MAE}}=\frac{1}{N}\sum_{i=1}^{N}\left|y_{i}-f(x_{i})\right|$$

(2) Root Mean Square Error (RMSE)

RMSE is the square root of the average of squared errors, which is more sensitive to larger errors in predictions and can effectively measure prediction accuracy and stability.

(45) $$V_{RMSE}={\left[\frac{1}{N}\sum_{i=1}^{N}{\left(y_{i}-f\left(x_{i}\right)\right)}^{2}\right]}^{1/2}$$

(3) Coefficient of Determination (R^2^)

R^2^ measures the proportion of variance in the observed data that is explained by the predicted results. A value closer to 1 indicates a better goodness-of-fit of the model to the system dynamics.

(46) $$R^{2}=1-\frac{\sum_{i=1}^{N}{\left(y_{i}-f(x_{i})\right)}^{2}}{\sum_{i=1}^{N}{\left(y_{i}-f(x_{i})\right)}^{2}}$$

(4) Dynamic Time Warping (DTW)

DTW calculates the minimum cumulative distance by finding the optimal nonlinear alignment between two time series, making it particularly suitable for evaluating the shape similarity of transient process curves that may have phase differences in the time axis.

(47) DTW(X,Y)=D(n,m)

where N is the total number of time steps; $y_{i}$ represents the actual value at the i-th time step; $f(x_{i})$ denotes the predicted value at the i-th time step, where DTW(X,Y) represents the Dynamic Time Warping distance between sequence X and sequence Y; D is the cumulative distance matrix; the length of X is n, and the length of Y is m.

### 5.3. Prediction Performance Analysis

**Figure 11:**
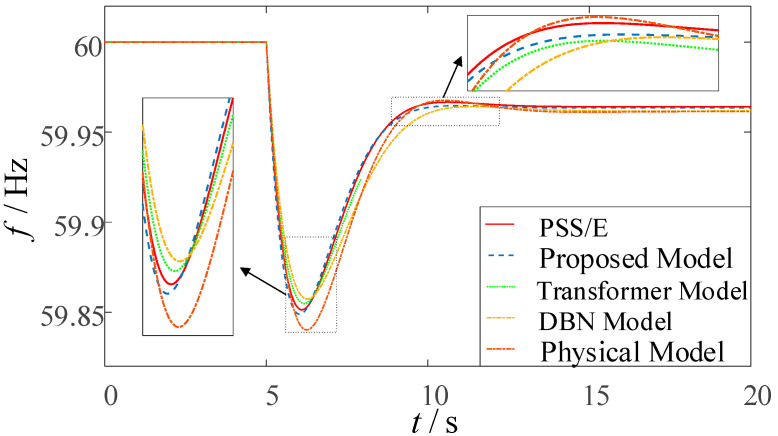
Comparison of transient frequency response curves under different prediction methods.

In order to verify the effectiveness of the method proposed in this paper, for the method in this paper, the pure physical model prediction method, the pure MLP-GRU-Attention model prediction method, the existing DBN model prediction method and the PSS/E time-domain simulation method, a sample is randomly selected among the test samples to carry out a comparative analysis of prediction effects under different methods, and the corresponding prediction results of its transient frequency are as follows The corresponding prediction results of transient frequency are shown in Figure 11.

As illustrated in Figure 11, the transient frequency response curve predicted by the proposed method demonstrates the closest agreement with the benchmark PSS/E simulation curve, accurately reproducing the complete dynamic process of frequency decline and recovery. During the initial stage following the disturbance, the transient frequency response curves predicted by all methods remain relatively similar. However, as time progresses, the frequency curves exhibit significant variations, and the discrepancies between the predictions of different methods become more pronounced. To evaluate the predictive performance of each method regarding key frequency response characteristics, a comparative statistical analysis of these key parameters was conducted, and the Absolute Errors (AE) were calculated, as summarized in Table 2 below.

As shown in Table 2, regarding the RoCoF, frequency nadir, time to reach frequency nadir, and steady-state frequency, the prediction accuracy of the proposed method for RoCoF shows improvements of 55.56%, 0%, and 63.6% compared to the purely MLP-GRU-Attention-based model, the purely DBN-based method, and the purely physics-based method, respectively. The prediction accuracy for frequency nadir is improved by 57.14%, 75%, and 98.9%; for the time to reach frequency nadir by 12.50%, 66.67%, and 68.18%; and for steady-state frequency by 80.00%, 50.00%, and 66.67%, respectively. These results demonstrate that the proposed method not only exhibits overall superior prediction performance compared to conventional approaches but also achieves higher accuracy in predicting key frequency characteristics. By integrating frequency response features with precisely measured data, the method achieves outstanding prediction precision. In particular, the RoCoF derived from the frequency response ensemble model effectively guides the model in capturing early-stage frequency dynamics, while the incorporated physical constraints limit the deviation between the predicted nadir and steady-state frequency within 0.003 Hz, further ensuring the reliability of the prediction results.

**Figure 12:**
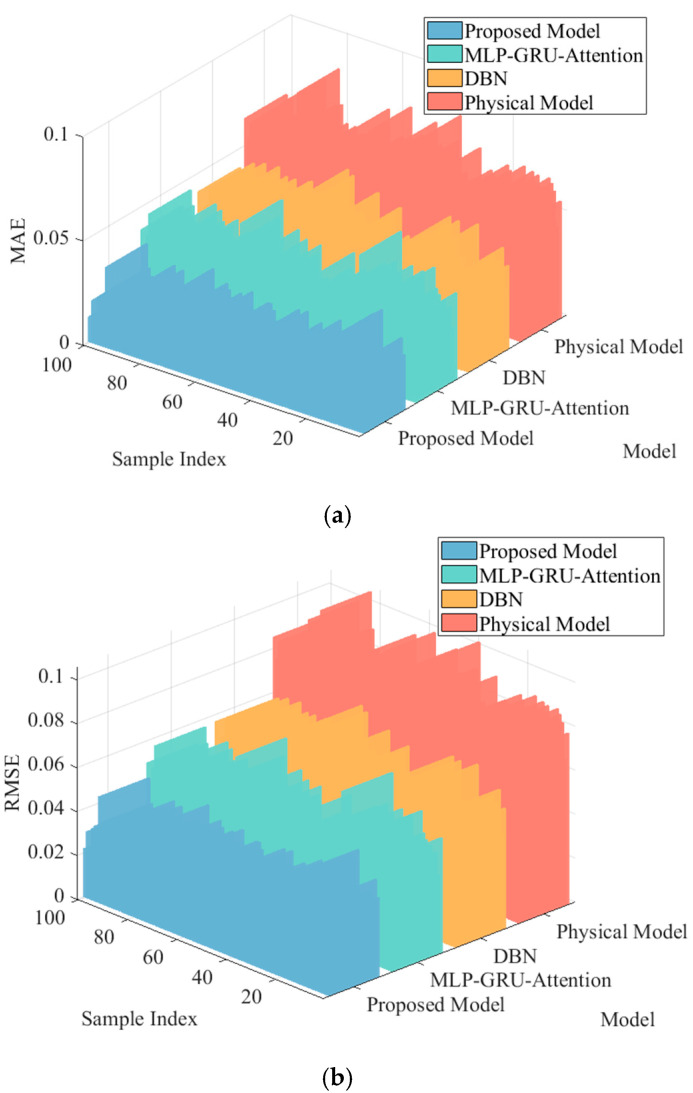

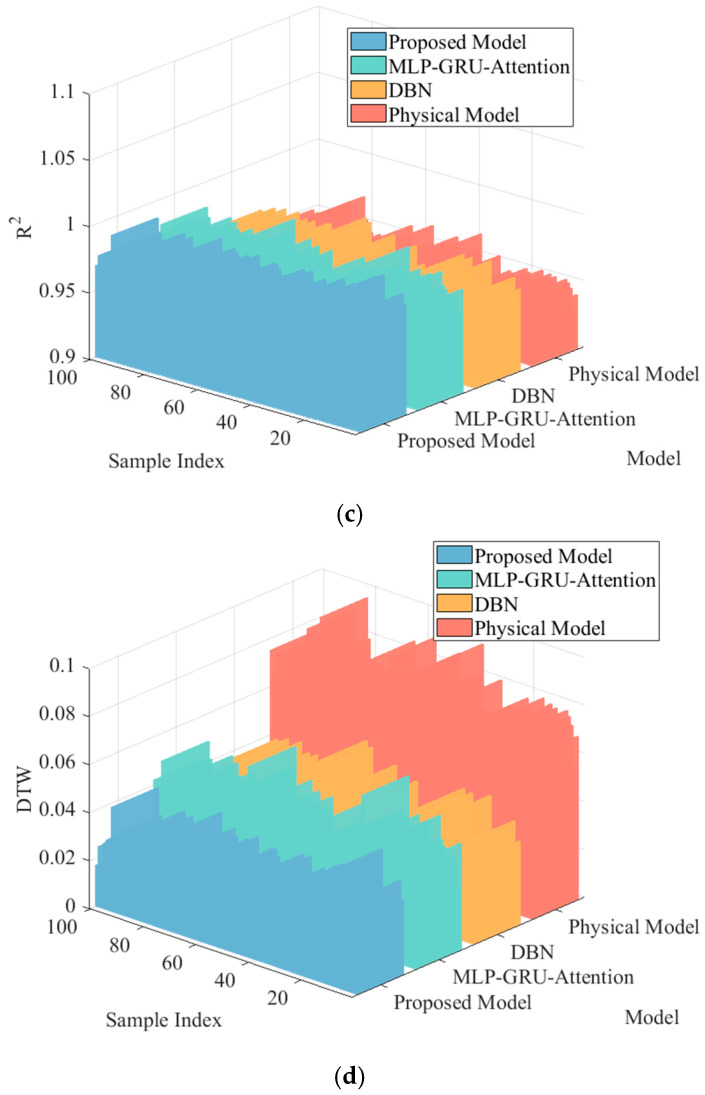
Comparison of model prediction performance of different methods on a random test sample set. (**a**) Comparison of MAE indicators. (**b**) Comparison of RMSE indicators. (**c**) Comparison of R^2^ indicators. (**d**) Comparison of DTW indicators.

To thoroughly validate the generalizability of the conclusions drawn from the aforementioned case analysis, the proposed fusion model, the standalone MLP-GRU-Attention model, and the DBN model were trained using the training dataset. Subsequently, 100 test samples were randomly selected from the original test set to evaluate the performance of each model in predicting post-disturbance transient frequency, with the computational results from the physical model also included for comparison. All predictive performance assessments were benchmarked against the PSS/E time-domain simulation results described in Section 5.1. Based on this benchmark, the average values of four metrics—RMSE, MAE, R^2^, and DTW—across all samples were calculated. The comprehensive predictive performance comparison of the four methods is ultimately presented in Figure 12.

As observed in Figure 12, the proposed method consistently demonstrates superior comprehensive performance across all four core evaluation metrics when benchmarked against PSS/E time-domain simulation results. The simultaneous significant reductions in both MAE and RMSE substantiate enhanced prediction accuracy from two perspectives: measuring average absolute deviation and penalizing large errors. Particularly, the minimized DTW distance indicates that the frequency curve predicted by our method exhibits the closest dynamic morphology to the actual curve, accurately capturing the temporal characteristics of the transient process. Although the absolute improvement in R^2^ appears modest, its value being closest to unity reflects the model’s optimal goodness-of-fit in representing the overall variation trend of frequency response, revealing its exceptional capability in capturing underlying physical mechanisms. These metrics collectively validate the comprehensive superiority of our fusion approach through multiple complementary dimensions, including error magnitude, curve morphology, and trend fitting.

### 5.4. Evaluation of Model Generalization Ability for Small Samples

**Figure 13:**
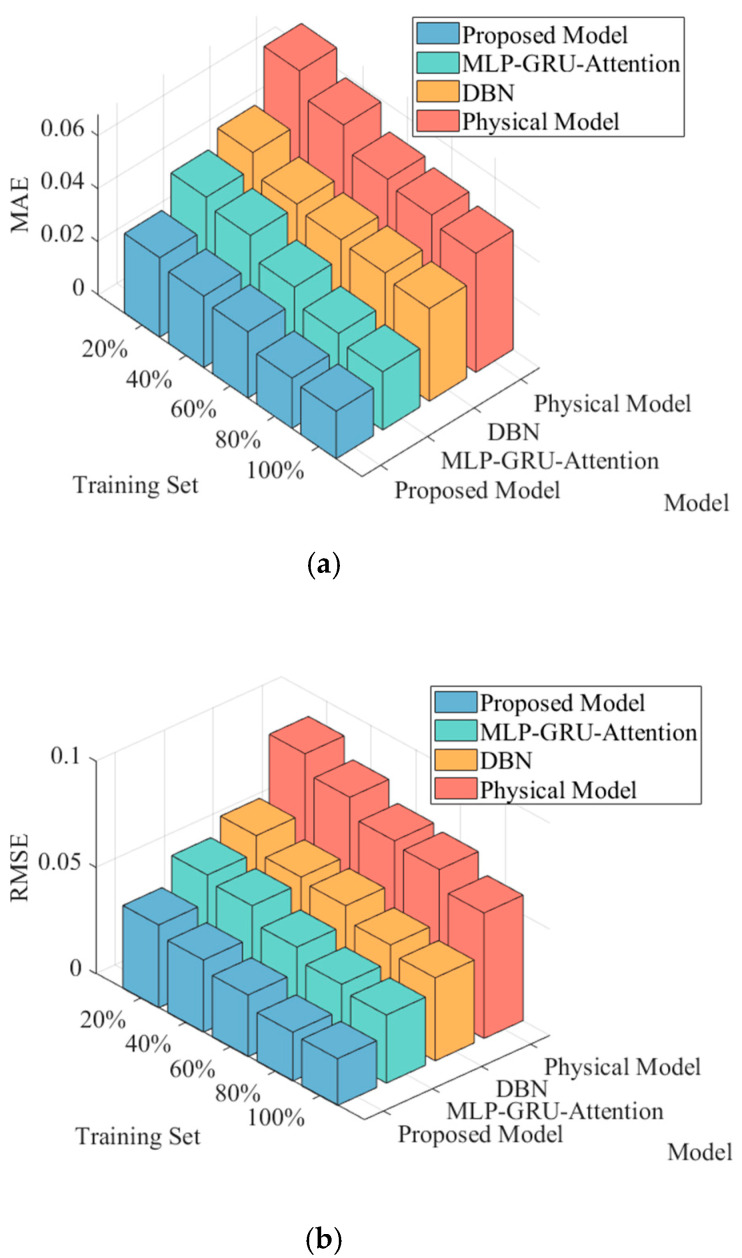

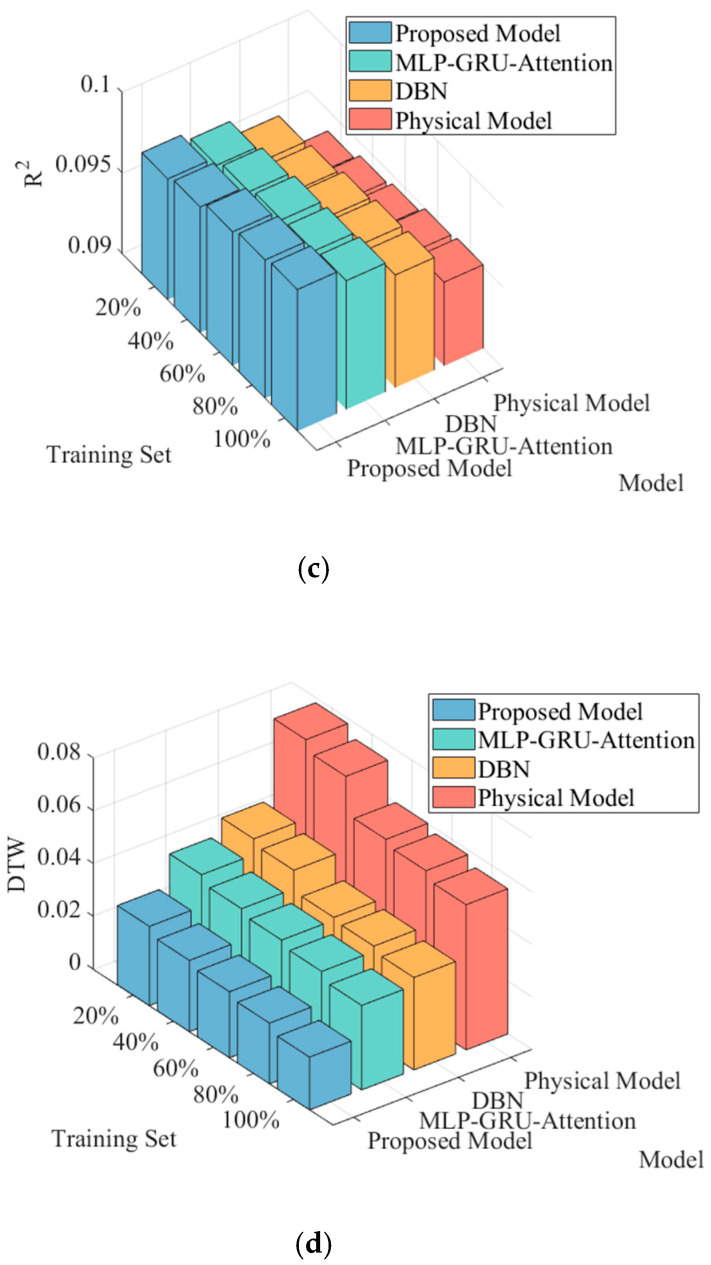
Comparison of model prediction performance of different methods under different training sets. (**a**) Comparison of MAE indicators. (**b**) Comparison of RMSE indicators. (**c**) Comparison of R^2^ indicators. (**d**) Comparison of DTW indicators.

In order to verify the generalization ability of the data-model fusion method proposed in this paper, 20%, 40%, 60%, 80%, and 100% of the total training samples are randomly selected as five new training sample sets, and the models of this paper’s method, MLP-GRU-Attention model prediction method, DBN model prediction method, and the physical model method are constructed for the comparative analysis of the prediction effect under the five training sets. The final comparison of the prediction effect of the four methods is shown in Figure 13.

As observed in Figure 13, with the increase in training sample size, the prediction error metrics of all models show a decreasing trend. Even under the limited 20% training sample condition, the proposed method maintains relatively lower values in MAE, RMSE, and DTW metrics with the smallest performance degradation, highlighting the effective enhancement of model generalization capability in small-sample scenarios through physics-informed knowledge embedding. This phenomenon can be attributed to the physical guidance mechanism providing prior knowledge to the model, reducing its dependency on pure data volume, thereby enabling effective learning of complex nonlinear mapping relationships even with scarce data. In summary, the proposed model demonstrates superior prediction accuracy and data utilization efficiency across all four core evaluation metrics, with these advantages becoming particularly prominent under insufficient training sample conditions.

### 5.5. Model Noise Resistance Evaluation

**Figure 14:**
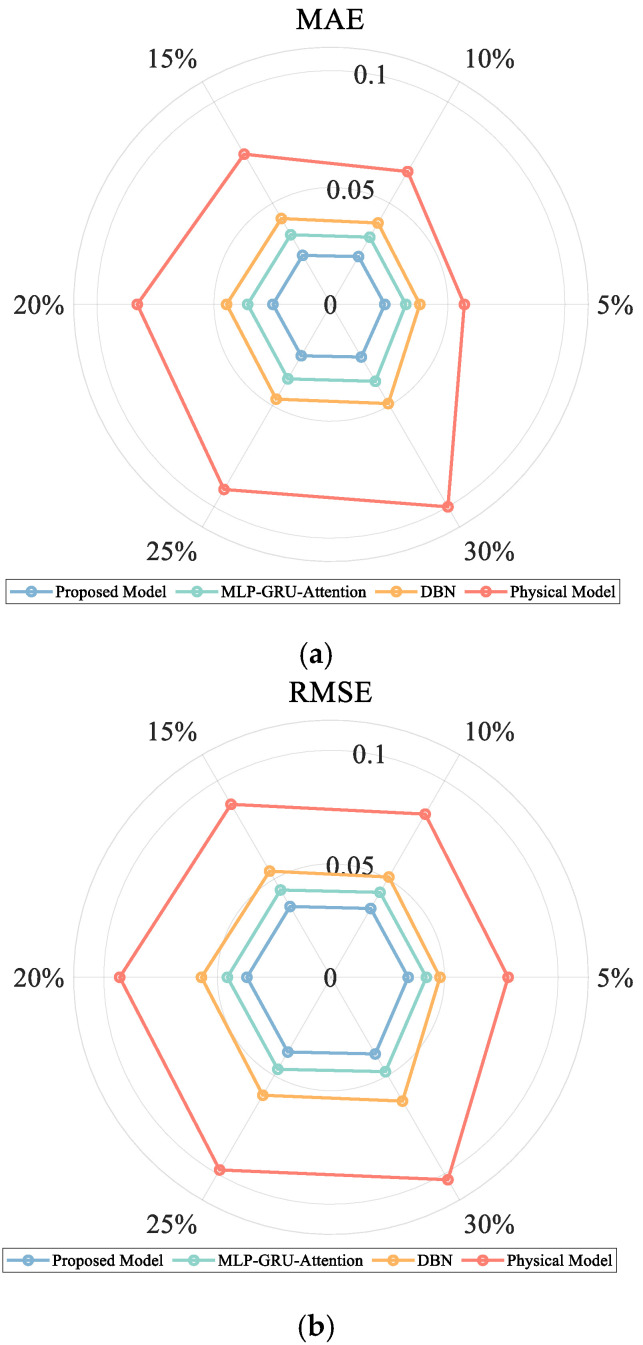

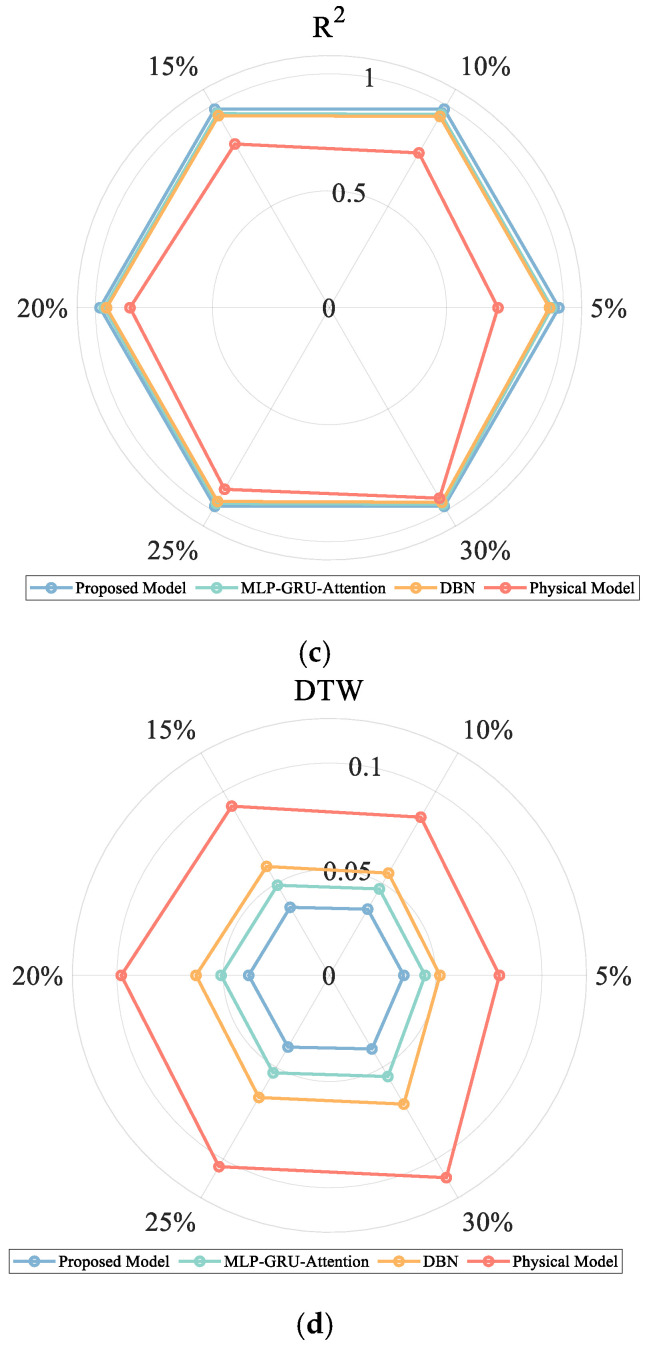
Comparison of model prediction performance of different methods under different Gaussian noise effects. (**a**) Comparison of MAE indicators. (**b**) Comparison of RMSE indicators. (**c**) Comparison of R^2^ indicators. (**d**) Comparison of DTW indicators.

Considering the actual engineering applications, the measurement data set of WAMS inevitably contains Gaussian white noise, impulse interference, and other composite error sources due to the limitation of sensor accuracy and the influence of communication channel interference in the data acquisition process. In order to evaluate the noise immunity of the model, this paper adds 5%, 10%, 15%, 20%, 25% and 30% Gaussian noise signals to the original test sample time series data to simulate the actual situation of WAMS measurement data. In order to verify the effectiveness of this paper’s method, it is compared with the purely physical model prediction method, the pure MLP-GRU-Attention model prediction method, and the existing DBN model prediction method under different noise levels, and the results of the evaluation index comparison are shown in Figure 14 below.

As shown in Figure 14, the proposed method demonstrates the most gradual increase in all evaluation metrics under progressively increasing noise levels. The slow growth of both MAE and RMSE quantitatively confirms the method’s insensitivity to measurement noise in terms of absolute error and prediction volatility, indicating excellent robustness. In contrast, traditional physics-based methods exhibit significantly rising evaluation metrics with increasing noise, primarily because their equivalent parameters are directly calculated from noisy data without establishing noise compensation mechanisms. The strong noise resistance of our method can be attributed to two key aspects: first, the data-driven module inherently possesses certain noise-smoothing capabilities; second, and more importantly, the embedded physics-guided framework enhances the contribution of key physical features through information entropy weighting and constrains prediction trajectories via physical consistency loss. This essentially enables adaptive noise filtering and trajectory calibration, thereby significantly improving reliability in challenging measurement environments.

## 6. Conclusions

Based on WAMS technology, this paper proposes a transient frequency response online prediction method that deeply integrates the WAMS data-driven model with the physical model. By constructing a physical model of frequency response that accounts for multiple types of FM resources, key features with clear physical significance are extracted; information entropy theory is introduced to realize adaptive weighted fusion of physical features; a physically guided MLP-GRU-Attention prediction framework is designed, and physical consistency constraints are introduced into the loss function to ensure that the prediction results are strictly in line with the dynamic laws of the system. Simulation validation based on the improved IEEE39-node system shows that the proposed method significantly outperforms the traditional data-driven and purely physical model methods in terms of prediction accuracy, generalization ability, and anti-noise performance, and the conclusions are as follows:

(1) The physics–data fusion method proposed in this paper effectively integrates the mechanistic interpretability of physical models with the high-precision learning capability of data-driven approaches through information entropy-weighted fusion of system-level key features extracted from the average frequency model and multi-bus measurement data provided by WAMS. As evidenced by simulation results, the proposed method demonstrates highly accurate reproduction of actual system frequency dynamics benchmarked against PSS/E simulations, exhibiting superior accuracy and reliability throughout the entire transient frequency response prediction process.
(2) By introducing the information entropy-weighted physical feature fusion and the physics-guided machine learning framework, the generalization ability of the model under small sample conditions is significantly improved, and its applicability in extreme disturbance scenarios is enhanced.
(3) The proposed method shows good robustness in the presence of WAMS measurement noise, and the embedding of physical knowledge effectively suppresses noise interference, maintains the stability and consistency of the prediction results, and provides reliable technical support for the online sensing and control of frequency security in new energy power grids.

## Figures and Tables

**Table 1:** Frequency response transfer function of various FM resources.

Control Measures	Auxiliary Power Transfer Function
Synchronous Generator Governor	$G_{m}=\frac{\Delta P_{m}}{\Delta f_{i}}=\frac{K_{m}(1+F_{H}T_{R}s)}{R(1+T_{R}s)}$
Virtual Inertia Control for Fans	$G_{\mathrm{wl}}=\frac{\Delta P_{\mathrm{wl}}}{\Delta f_{i}}=\frac{k_{d}s+k_{p}}{T_{\mathrm{wl}}s+1}$
Photovoltaic and Energy Storage Droop Control	$G_{\mathrm{el}}=\frac{\Delta P_{\mathrm{el}}}{\Delta f_{i}}=\frac{k_{\mathrm{elp}}}{T_{\mathrm{el}}s+1}$
DC Transmission FLC	$G_{\mathrm{DC}}=\frac{\Delta P_{\mathrm{DC}}}{\Delta f_{i}}=\frac{k_{\mathrm{DCP}}s+k_{I}}{T_{\mathrm{DC}}s^{2}+s}$

**Table 2:** Comparison of key transient frequency characteristics under different prediction methods.

Methodologies	Initial Frequency Rate of Change (Hz/s)		Minimum Frequency (Hz)		Minimum Frequency Arrival Time (s)		Steady State Frequency (Hz)	
	$\Delta f_{\mathrm{begin}}$	AE	$\Delta f_{\min}$	AE	$t_{z}$	AE	$f_{\mathrm{ss}}$	AE
Methodology of this paper	0.124	0.004	59.849	0.003	6.02	0.07	59.963	0.001
MLP-GRU-Attention modeling approach	0.111	0.009	59.859	0.007	6.17	0.08	59.959	0.005
DBN modeling approach	0.116	0.004	58.864	0.012	6.30	0.21	59.962	0.002
physical model	0.131	0.011	59.836	0.016	6.31	0.22	59.961	0.003

## Data Availability

Data is contained within the article.

## Abbreviations

The following abbreviations are used in this manuscript:

FM	Frequency Modulation
SFR	System Frequency Response
WAMS	Wide-Area Measurement System
SME	Single-Machine Equivalent
HVDC	High Voltage Direct Current Transmission
RoCoF	Rate of Change of Frequency
UFLS	Under-Frequency Load Shedding
FLC	Frequency Limiting Controller
PI	Proportional-Integral
PV	Photovoltaic
MLP	Multilayer Perceptron
GRU	Gated Recurrent Unit
DBN	Deep Belief Network
RNN	Recurrent Neural Network
1D-CNN	One-Dimensional Convolutional Neural Network
TFAM	Temporal-Feature Attention Module
FSPM	Frequency Security Predictor Model
FDL	Frequency Danger Level
TSM	Time Security Margin
LSTM	Long Short-Term Memory
WOA	Whale Optimization Algorithm
NAR	Nonlinear AutoRegressive
MSE	Mean Squared Error
MAE	Mean Absolute Error
RMSE	Root Mean Square Error
MRE	Mean Relative Error
R^2^	Coefficient of Determination
DTW	Dynamic Time Warping
PMU	Phasor Measurement Unit
